# Karyotypic features including organizations of the 5S, 45S rDNA loci and telomeres of *Scadoxus multiflorus* (Amaryllidaceae)

**DOI:** 10.3897/CompCytogen.v10i4.9958

**Published:** 2016-11-22

**Authors:** Pansa Monkheang, Arunrat Chaveerach, Runglawan Sudmoon, Tawatchai Tanee

**Affiliations:** 1 Department of Biology, Faculty of Science, Khon Kaen University, Khon Kaen 40002, Thailand; 2 Genetics and Environmental Toxicology Research Group, Khon Kaen University, Khon Kaen 40002, Thailand; 3 Faculty of Law, Khon Kaen University, Khon Kaen 40002, Thailand; 4 Faculty of Environment and Resource Studies, Mahasarakham University, Maha Sarakham 44150, Thailand

**Keywords:** Scadoxus multiflorus, aceto-orcein staining, fluorescence *in situ* hybridization (FISH), rDNA, telomere

## Abstract

*Scadoxus multiflorus* Martyn, 1795 is an ornamental plant with brilliantly colored flowers. Even though its chromosomes are rather large, there is no karyotype description reported so far. Therefore, conventional and molecular cytogenetic studies including fluorescence *in situ* hybridization (FISH) with 45S and 5S rDNA, and human telomere sequence (TTAGGG)_n_ probes (Arabidopsis-type telomere probes yielded negative results) were carried out. The chromosome number is as reported previously, 2n = 18. The nine chromosome pairs include two large submetacentric, five large acrocentric, one medium acrocentric, two small metacentric and eight small submetacentric chromosomes. Hybridization sites of the 45S rDNA signals were on the short arm ends of chromosomes #1, #3 and #8, while 5S rDNA signals appeared on the long arm of chromosome 3, in one homologue as a double signal. The telomere signals were restricted to all chromosome ends. Three chromosome pairs could be newly identified, chromosome pair 3 by 5S rDNA and chromosomes #1, #3 and #8 by 45S rDNA loci. In addition to new information about rDNA locations we show that the ends of *Scadoxus multiflorus* chromosomes harbor human instead of Arabidopsis-type telomere sequences. Overall, the *Scadoxus multiflorus* karyotype presents chromosomal heteromorphy concerning size, shape and 45S and 5S rDNA positioning. As *Scadoxus* Rafinesque, 1838 and related species are poorly studied on chromosomal level the here presented data is important for better understanding of evolution in Amaryllidaceae.

## Introduction

*Scadoxus multiflorus* Martyn, 1795 (also known as *Haemanthus multiflorus* Martyn, 1795 and *Haemanthus kalbreyeri* Baker, 1878) is a species belonging to the family Amaryllidaceae (Chase et al. 2009), which can naturally only be found in Southern and tropical Africa (Patwary and Zaman 1980). Formerly, the genus *Scadoxus* Rafinesque, 1838 was *Haemanthus* Linnaeus, 1753 and was included in the family Liliaceae (Ahirwar and Verma 2014); however, it is not included in the current circumscription of this family (Peruzzi 2016), and was indeed separated out and relocated by Angiosperm Phylogeny Group (APG IV) classification system.

*Scadoxus multiflorus* is economically important as it is popular as cultivated ornamental plant. Nonetheless, this species was not studied yet for its karyotype in details. There are only two previous reports based on conventional chromosome staining of *Scadoxus multiflorus*. Both studies reported 2n = 18 chromosomes, however, Ahirwar and Verma (2014) found six submetacentric and 12 acrocentric chromosomes while Patwary and Zaman (1980) reported one metacentric, nine submetacentric, six acrocentric and two telocentric chromosomes. Besides these contradictory data, molecular cytogenetic approaches like florescence *in situ* hybridization (FISH) have not been applied in this species yet. Such methods being available since the late 1980s (Li et al. 2016, Taguchi et al. 2016) enable detection, characterization and localization of rDNA regions (Chirino et al. 2015) and/or telomeres. The latter are known to be important to protect chromosomal ends of all eukaryotes against nucleolytic degradation, non-homologous end-joining and replication-mediated shortening. They usually consist of short tandem repeats, such as (TTTAGGG)_n_ in *Arabidopsis* Heynhold, 1842 (Richards and Ausubel 1988) or (TTAGGG)_n_ in humans (Moyzis et al. 1988). The *Arabidopsis* G-rich telomere repeat is rather conserved and has been detected at the ends of most chromosomes of higher plants examined so far. Nevertheless, in some plants the TTTAGGG-type telomere repeat is lacking and substituted by other repeat sequences (Fajkus et al. 2016).

As for *Scadoxus multiflorus* reported karyotypic details are contradictory and no FISH studies have been performed so far the present study aimed to close this gap of knowledge using conventional staining and FISH.

## Material and methods

### Plant material and chromosome preparation

*Scadoxus multiflorus* plant was collected in Khon Kaen Province, Northeastern, Thailand (A. Chaveerach et al. 903, Department of Biology, Faculty of Science, Khon Kaen University, Khon Kaen, Thailand). The roots were collected from bulbs placed in distilled water at room temperature. The root tips were excised and kept in cold water for 1 h at 4°C, after that transferred to 0.05% colchicine solution for 4 h at room temperature to accumulate metaphase chromosomes before fixation in ethanol:acetic acid (3:1, v/v) for at least 24 h at 4°C. The protocol for the SteamDrop method (Kirov et al. 2014) was adopted with a few modifications. Briefly, fixed root tips were washed twice with enzyme buffer (0.01 M citric acid, 0.01 M sodium citrate, pH 4.7) to remove the fixative and digested at 37°C for 4 h in enzyme solution consisting of 0.7% cellulase R10 (Duchefa C8001), 0.7% cellulase (CalBioChem 319466), 1% pectolyase (Sigma P3026) and 1% cytohelicase (Sigma C8274) in enzyme buffer. Then, the soft meristematic tissues were washed twice with distilled water and 96% ethanol to remove supernatant with centrifuge before broken with a dissecting needle in a tube in fixative. The suspension was dropped on a glass slide and air dried. Preparations with well spread metaphases were selected for further analyses.

### Orcein staining and idiogram generation

Conventional staining was carried out on slides using 2% aceto-orcein for 5 min at room temperature and then covered with a coverslip. Ten well-spread metaphases were selected for photomicrography with a digital camera under oil immersion by light microscope. The length of short and long chromosome arms (p and q) were measured separately and added to calculate the total length (LT). The relative length of chromosome (RL), the centromeric index (CI) and standard deviation (SD) of RL and CI were calculated according to Chaiyasut (1989). The CI (q/p+q) between 0.500–0.599, 0.600–0.699, 0.700–0.899 and 0.900–0.999 were considered as metacentric (m), submetacentric (sm), acrocentric (a) and telocentric (t), respectively, following Turpin and Lejeune (1965) to classify the types of chromosome. These parameters were used for idiograming by computer. Comparison of different estimators of intrachromosomal asymmetry was performed, including mean centromeric asymmetry (M_CA_) and coefficient of variation of chromosome length (CV_CL_) based on the equations provided by Peruzzi and Eroğlu (2013). Metaphase chromosomes from overall 10 cells were included.

### DNA probe generation and labeling

45S and 5S rDNAs which were isolated from *Arabidopsis thaliana* Schur, 1866 and telomere repeat sequences from *Arabidopsis* (TTTAGGG)_n_ and human (TTAGGG)_n_ were applied in this study. The plasmid of 45S rDNA cloned in the vector T_15_P_10_IV_1_ was labelled with Alexa 488-dUTP, while the 5S rDNA probe was labelled with Cy3-dUTP by Nick translation (Roche Cat No 11745808910). The telomeric probes were generated by polymerase chain reaction (PCR) in absence of a DNA template using primers (TTTAGGG)_5_ and (CCCTAAA)_5_, and (TTAGGG)_5_ and (CCCTAA)_5_ according to Ijdo et al. (1991) and labelled with Cy3-dUTP by nick translation.

### Fluorescence *in situ* hybridization

A FISH protocol according to Lysak et al. (2006) was applied with minor modifications. The slide with fixed metaphase cells was washed in 2 × SSC (300 mM Na-citrate, 30 mM NaCl, pH 7.0) for 5 min at room temperature and treated with 45% acetic acid for 3 min. Then the slides were washed twice in 2 × SSC for 5 min each at room temperature before digestion in pepsin solution (10 mg/ml) in 0.01 M HCl for 1 min at 37°C, rinsing twice in 2 × SSC for 5 min, post-fixation in 4% formaldehyde in 2 × SSC for 5 min at room temperature, and two washes in 2 × SSC, with final dehydration in an ethanol series (70%, 90%, 100%) for 2 min, each, at room temperature and air drying.

A 3 µl (60 ng) of labelled probe was dissolved in 17 µl of hybridization mixture (20% dextran sulfate, 50% formamide in 2 × SSC, pH 7.0), and pre-denatured at 95°C for 5 min. Then the solution was added to the slide, covered with a coverslip and sealed by rubber gum. Now, the slide was placed on a heating plate at 80°C for 2 min for co-denaturation of probe and target DNA and incubated in a moist chamber at 37°C for 18 h for hybridization.

After hybridization, slides were washed in 2 × SSC for 5 min at 42°C and three times with 50% formamide in 2 × SSC for 5 min at 42°C. After that, slides were washed three times with 2 × SSC for 5 min at 42°C for 5 min each. Finally, the slides were dehydrated in an ethanol series, air dried, counterstained with 4, 6-diamino-2-phenylindole (DAPI) plus Vectashield antifade mounting medium (Vector Laboratories, USA) and covered with a coverslip. Signals were detected using an epifluorescence microscope with Triple filter (UV, Texas Red and FITC) and photographed (microscope: Axioplan2, Zeiss; Camera: Hammamatsu-ORCA-ER C4742-80, Japan; Lamp: Flouarc, Zeiss).

## Results

**Figure 1.**
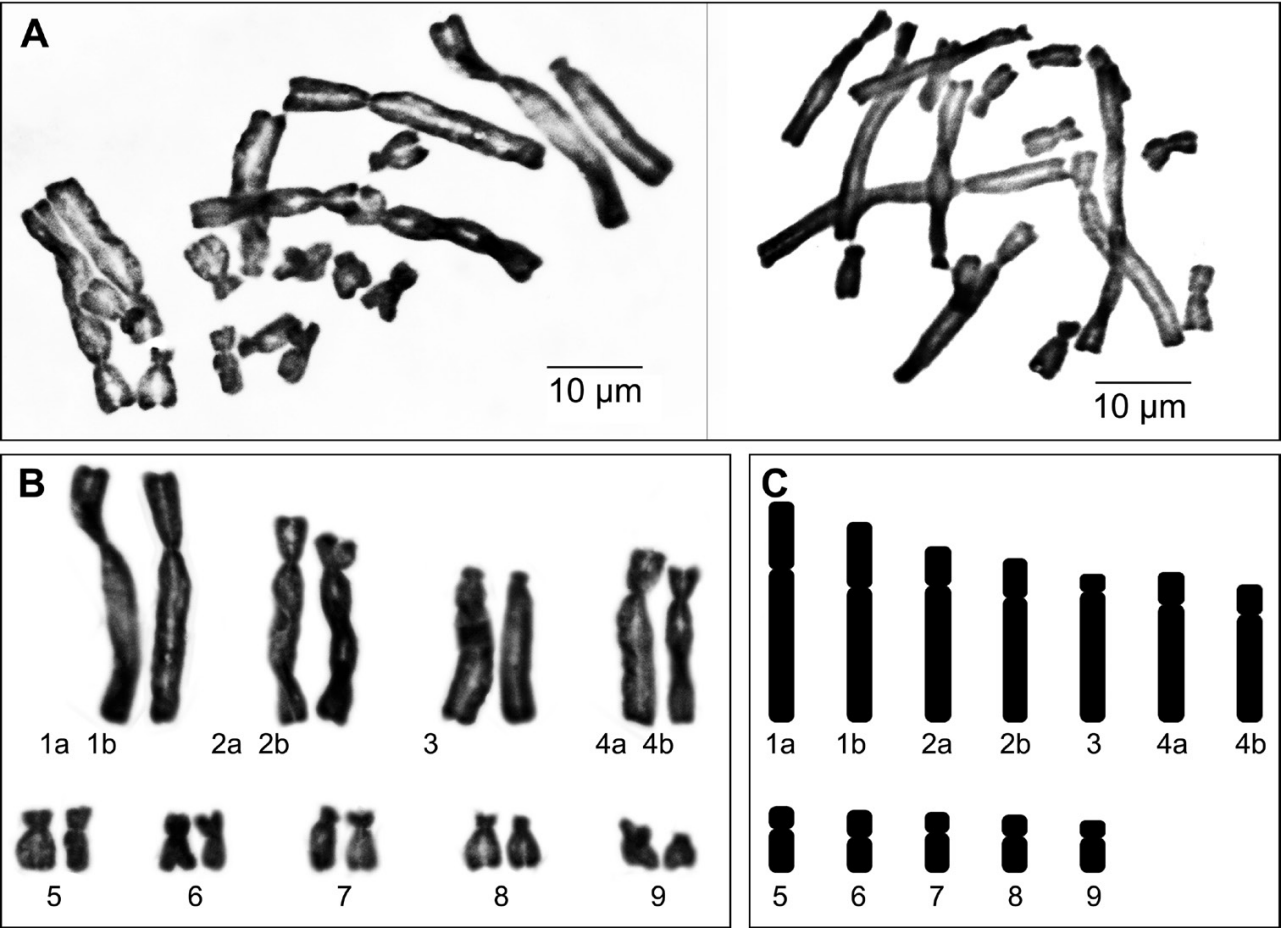
Mitotic metaphase chromosomes of *Scadoxus multiflorus* (2n = 18). **A** aceto-orcein staining of two cells **B** karyogram showing four large and five small pairs of chromosomes **C** idiogram.

**Figure 2.**
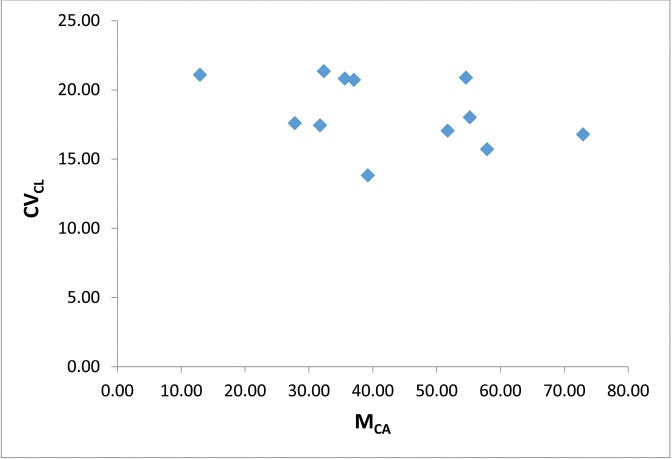
Scatter plot of M_CA_ against CV_CL_ of *Scadoxus multiflorus* chromosomes.

The idiogram and karyotype analyses established from the metaphases confirmed the diploid chromosome number of *Scadoxus multiflorus* to be 2n = 18. The karyotype analysis of this species is summarized in Table 1. The range of total arm length, centromeric index and relative chromosome length are 3.78–16.01 µm, 0.57–0.87 µm and 0.04–0.15 µm respectively. The karyotype comprises two large submetacentric, five large acrocentric, one medium acrocentric, two small metacentric and eight small submetacentric chromosomes (Fig. 1). The plant shows a clear tendency to have karyotypes distinct on asymmetry grounds with relatively low M_CA_ and CV_CL_ as shown in Figure 2 and Table 2.

**Table 1.** Mean length of the short arm chromosome (Ls), long arm chromosome (Ll), total arm chromosome (LT), centromeric index (CI), relative length (RL) and standard deviation (SD) of CI, RL from metaphase chromosomes in 10 cells of the blood lily (*Scadoxus multiflorus*), 2n = 18.

Chr.	Ls	Ll	LT	CI ± SD	RL ± SD	Type	Size
1a*	4.86	11.14	16.007	0.696 ± 0.068	0.148 ± 0.010	sm	L
1b*	4.66	9.81	14.465	0.678 ± 0.100	0.134 ± 0.025	sm	L
2a*	2.85	9.89	12.741	0.776 ± 0.085	0.118 ± 0.008	a	L
2b*	2.86	9.00	11.865	0.759 ± 0.073	0.110 ± 0.015	a	L
3	1.49	9.52	11.008	0.865 ± 0.131	0.102 ± 0.015	a	L
4a*	2.29	8.58	10.870	0.790 ± 0.087	0.101 ± 0.013	a	L
4b*	2.15	7.33	9.480	0.773 ± 0.074	0.088 ± 0.016	a	M
5	1.62	3.17	4.784	0.662 ± 0.066	0.044 ± 0.006	sm	S
6	1.95	2.53	4.485	0.565 ± 0.034	0.042 ± 0.004	m	S
7	1.50	2.90	4.402	0.659 ± 0.063	0.041 ± 0.004	sm	S
8	1.50	2.65	4.153	0.639 ± 0.065	0.038 ± 0.004	sm	S
9	1.19	2.59	3.779	0.685 ± 0.069	0.035 ± 0.005	sm	S

Remarks: Chr. = chromosome pair , a = acrocentric , m = metacentric , sm = submetacentric , L = large , M = medium , S = small , a* and b* = heteromorphic pairs 1, 2, and 4.

**Table 2.** Comparison of different estimators of intrachromosomal asymmetry including mean centromeric asymmetry (M_CA_) and coefficient of variation of chromosome length (CV_CL_) from metaphase chromosomes in 10 cells of the blood lily (*Scadoxus multiflorus*), 2n = 18.

Chr.	Mean from 10 metaphases						SD of chr. length	M_CA_	CV_CL_
	Ll-Ls	Ls Ll	Ls (Ll+Ls)	Ll (Ll+Ls)	(Ll-Ls) Ll	(Ll-Ls) (Ll+Ls)			
1a*	6.28	0.44	0.30	0.70	0.56	0.39	2.21	39.23	13.82
1b*	5.15	0.47	0.32	0.68	0.53	0.36	3.01	35.61	20.82
2a*	7.03	0.29	0.22	0.78	0.71	0.55	2.30	55.20	18.02
2b*	6.14	0.32	0.24	0.76	0.68	0.52	2.02	51.72	17.05
3	8.03	0.16	0.14	0.86	0.84	0.73	1.85	72.94	16.78
4a*	6.29	0.27	0.21	0.79	0.73	0.58	1.71	57.90	15.71
4b*	5.18	0.29	0.23	0.77	0.71	0.55	1.98	54.60	20.89
5	1.55	0.51	0.34	0.66	0.49	0.32	1.02	32.34	21.35
6	1.40	0.52	0.34	0.66	0.48	0.32	0.77	31.74	17.44
7	0.58	0.77	0.44	0.56	0.23	0.13	0.95	12.93	21.09
8	1.15	0.57	0.36	0.64	0.43	0.28	0.73	27.79	17.60
9	1.40	0.46	0.31	0.69	0.54	0.37	0.78	37.04	20.73

Remarks: Chr. = chromosome pair , a = acrocentric , m = metacentric , sm = submetacentric , L = large , M = medium , S = small , a* and b* = heteromorphic pairs 1, 2, and 4.

**Figure 3.**
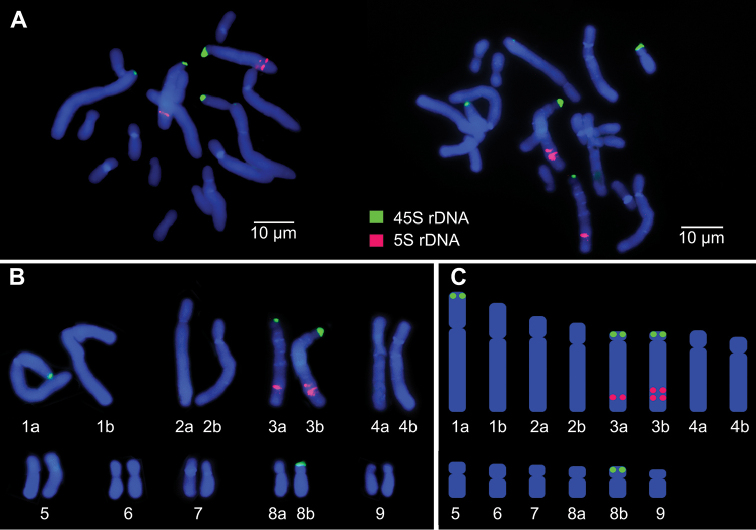
Organizations of 45S rDNA and 5S rDNA loci on metaphase chromosomes of *Scadoxus multiflorus* (2n = 18). **A** FISH signals of 45S (Alexa, green) and 5S (Cy3, red) rDNA probes on two cells **B** karyogram **C** idiogram.

The metaphase spreads were hybridized with 45S (Alexa, green) and 5S (Cy3, red) rDNA probes as shown in Fig. 3. The hybridization signals for the 45S rDNA probe are in terminal positions of the short arms of chromosomes #1, #3 and #8 (Fig. 3A). The 5S rDNA signals were detected on the long arms of chromosome #3 with one homologue showing two adjacent signals (Figs 3B–C).

**Figure 4.**
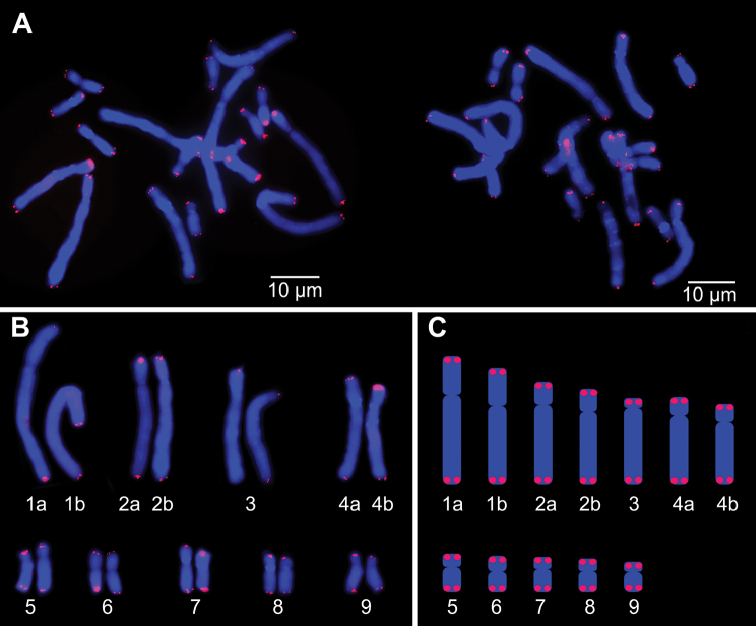
Localization of the human telomere repeat sequence (TTAGGG)_n_ on metaphase chromosomes of *Scadoxus multiflorus* (2n = 18). **A** FISH signals of the (TTAGGG)_n_ probe on two cells **B** karyogram **C** idiogram.

The hybridization of the Arabidopsis-type telomeric probe yielded no obvious FISH signal, while the human-type (Cy3, red) revealed small signals exclusively at the end of all *Scadoxus multiflorus* chromosomes (Fig. 4).

Overall, the results show chromosomal heteromorphy in sizes and shapes.

## Discussion

Here we provide the first study of *Scadoxus multiflorus* chromosomes by means of molecular cytogenetics. Furthermore, the previously reported chromosome number could be confirmed to be 2n = 18 (Patwary and Zaman 1980, Ahirwar and Verma 2014). However, here we report a karyotype variant in *Scadoxus multiflorus* as carrying two metacentric, ten submetacentric and six acrocentric chromosomes, which is in contradiction to what was previously reported by Patwary and Zaman (1980) or Ahirwar and Verma (2014). This result indicates that further studies are necessary to clarify if there are either (i) cryptic (sub-)species in *Scadoxus multiflorus*, (ii) assessment problems in any of the previous studies, or (iii) any kind of chromosomal heteromorphisms leading to the observed different chromosomal shapes.

Although the regions of 5S and 45S were not observed with conventional staining, due to several limitations, such as oil-immersion light microscopic methods, genetic processing and analysis standards, the signal intensities of rDNA probes in FISH showed clear variation in copy numbers. The copy number and distributed position on chromosome are very important as species markers (Boocock et al. 2015, Li et al. 2016).

In a group of families of the monocot order Asparagales, the telomeric sequence (TTAGGG)_n_ of the human-type was found to be maintained (Sykorova et al. 2003). Thus it is in accordance with the literature that *Scadoxus multiflorus* also has this type of telomeric repeat, as the family Amaryllidaceae belongs to this branch of Asparagales which switched from *Arabidopsis*-type to human type telomere sequence. The knowledge is the one factor supports the classification theory of a common ancestor for a plant group.

Overall, our results allow now distinguishing five of the nine *Scadoxus multiflorus* chromosome pairs individually. Development of suitable genomic single-copy FISH probes might allow discrimination of all chromosome pairs and to use them for identification of homologous chromosomes in other species of genus, or even of related genera. As *Scadoxus* and related species are poorly studied on chromosomal level the here presented data is important for better understanding of evolution in Amaryllidaceae.
